# 2023 Relationship power imbalance and history of male partner HIV testing among pregnant women in central Uganda

**DOI:** 10.1017/cts.2018.115

**Published:** 2018-11-21

**Authors:** Caroline Vrana, Jeffrey Korte, Angela Malek, Esther Buregyeya, Joseph Matovu, Harriet Chemusto, William Musoke, Rhoda Wanyenze

**Affiliations:** 1 Medical University of South Carolina; 2 Public Health Sciences, MUSC; 3 School of Public Health, Makerere University; 4 Mildmay Uganda

## Abstract

OBJECTIVES/SPECIFIC AIMS: We investigated the association between relationship power imbalance (which can have a negative impact on HIV prevention) and male partner HIV testing, using baseline data from a HIV self-testing trial in 3 antenatal clinics in central Uganda. METHODS/STUDY POPULATION: Pregnant women with HIV-male partners were recruited and randomized by day into standard of care or intervention (HIV self-testing kits). Analyses were performed in SAS 9.4, with χ^2^ tests and *p*<0.05 for significance. RESULTS/ANTICIPATED RESULTS: In total, 1514 women were recruited (737 standard of care, 777 intervention). Overall, 39.6% of male partners had previously tested for HIV. Among women <26, contributions to expenses differed by partner testing (overall *p*<0.001, 47.6% of women whose partners tested made no contribution vs. 63.2% of women whose partners did not test). Relationship status differed by partner testing (overall *p*=0.02, 12.4% of women whose partners tested showed a sometimes difficult relationship vs. 5.7% of women whose partners did not test). Among women 26+, decision making for family visits differed by partner testing (overall *p*=0.005, 52.9% of women made joint decisions with partners who tested vs. 36.5% whose partners did not test). DISCUSSION/SIGNIFICANCE OF IMPACT: Higher relationship power balance was associated with higher HIV testing among male partners when measured by contribution to expenses and decision making for family visits, but not relationship status. Relationship power balance should be considered when counseling women and men to increase HIV testing.

